# The role of immune cells in the pathogenesis of connective tissue diseases-associated pulmonary arterial hypertension

**DOI:** 10.3389/fimmu.2024.1464762

**Published:** 2024-09-17

**Authors:** Zhe Li, Juan Ma, Xuejing Wang, Liquan Zhu, Yu Gan, Baoquan Dai

**Affiliations:** ^1^ Department 5 of Pediatric, Weifang Maternal and Child Health Hospital, Weifang, China; ^2^ School of Rehabilitation Medicine, Shandong Second Medical University, Weifang, China

**Keywords:** inflammation, immunity, cytokines, chemokines, pulmonary hypertension, immunosuppressive therapy

## Abstract

Connective tissue diseases-related pulmonary arterial hypertension (CTD-PAH) is a disease characterized by an elevated pulmonary artery pressure that arises as a complication of connective tissue diseases. The number of patients with CTD-PAH accounts for 25.3% of all PAH patients. The main pathological features of CTD-PAH are thickening of intima, media and adventitia of pulmonary arterioles, increased pulmonary vascular resistance, autoimmune activation and inflammatory reaction. It is worth noting that abnormal immune activation will produce autoantibodies and release cytokines, and abnormal immune cell recruitment will promote inflammatory environment and vascular remodeling. Therefore, almost all forms of connective tissue diseases are related to PAH. In addition to general therapy and targeted drug therapy for PAH, high-dose glucocorticoid combined with immunosuppressant can quickly alleviate and stabilize the basic CTD-PAH disease. Given this, the development of therapeutic approaches targeting immune dysregulation and heightened inflammation is recognized as a promising strategy to prevent or reverse the progression of CTD-PAH. This review explores the potential mechanisms by which immune cells contribute to the development of CTD-PAH and examines the clinical application of immunosuppressive therapies in managing CTD-PAH.

## 1 Introduction

Connective tissue disease-associated pulmonary arterial hypertension (CTD-PAH) refers to the progressive elevation of pulmonary artery caused by connective tissue disease, which is a common complication of CTD (1, 2). CTD-PAH belongs to the category of PAH. CTD-PAH patients account for 25.3% of all PAH patients, and it is the second most common cause of PAH, second only to the idiopathic form. The pathological features of CTD-PAH are mainly pulmonary vascular remodeling, including pulmonary arteriole middle-layer hypertrophy, intimal fibrosis, plexiform lesions and tiny pulmonary artery occlusion, etc (3, 4). These changes are closely related to vascular endothelial injury, proliferation and migration of smooth muscle cells (SMCs), extracellular matrix deposition and chronic inflammatory reaction mediated by immune abnormalities, which together lead to increased pulmonary artery pressure and increased pulmonary circulation resistance (5, 6). Among them, immune imbalance is an important feature of CTD-PAH, which is very important for the initiation and maintenance of vascular remodeling (7). For example, the change of vascular cell phenotype leads to the change of sensitivity to inflammatory trigger, the enhancement of self-staged inflammatory response and the active secretion of cytokines and chemokines (8). At present, in clinical treatment, in addition to general treatment and targeted drug therapy for PAH, high-dose glucocorticoid combined with immunosuppressant can quickly alleviate and stabilize the basic CTD condition, and can effectively improve CTD-PAH (9).

CTD encompass a broad range of systemic autoimmune rheumatic conditions that affect multiple organ systems, such as systemic lupus erythematosus related PAH (SLE-PAH), systemic sclerosis related PAH (SSc-PAH), connective tissue disease related PAH (MCTD-PAH), and rheumatoid arthritis related PAH, etc (10, 11). CTD-PAH is different in different regions. For example, in Europe and the United States, systemic sclerosis is the main cause, while in Asia, systemic lupus erythematosus is more common (11). Notably, patients with SLE-PAH tend to respond more favorably to treatm (12–14). These conditions are characterized by immune dysregulation and the production of disease-specific autoantibodies (15, 16). In addition, the pathogenesis of these diseases involves immunity and vascular remodeling. In patients with CTD-PAH, antibodies and immune complexes are often deposited on the pulmonary artery wall, especially anti-U1RNP antibodies (17). These antibodies can significantly up-regulate the expression of adhesion factors (such as ICAM21 and ELAM21) and MHC class II molecules in pulmonary artery endothelial cells (ECs), leading to inflammatory cells infiltrating the vascular wall. The deposition of immune complex will attract inflammatory cells (such as neutrophils, macrophages, etc.) to infiltrate into the blood vessel wall, causing vasculitis and cellulose necrosis, further aggravating blood vessel injury (6). The autoimmune reaction of CTD-PAH patients is extremely active, which leads to inflammatory reaction and fibrosis changes in pulmonary vascular wall, which is an important basis for the formation of pulmonary hypertension (6). Due to the infiltration of inflammatory cells and the deposition of immune complexes, the inner ECs are damaged, resulting in intima thickening. Stimulated by inflammatory factors, vascular SMCs will proliferate abnormally, aggravating lumen stenosis (18). So, this paper reviews the potential pathogenesis of CTD-PAH in autoimmune and immune dysregulation in recent years. And further put forward the feasibility of immunosuppressive treatment strategy in CTD-PAH.

## 2 Immune activation in CTD-PAH

The pathogenesis of CTD-PAH is intricate and not fully elucidated. Currently, the predominant theory associates PAH with extensive vascular remodeling (19). Its main characteristics are proliferation of ECs and SMCs, fibrinoid necrosis caused by vasculitis, and deposition of immunoglobulin and complement components in intima and medial layers of pulmonary blood vessels (20). Under normal conditions, blood vessels maintain a balanced state between constriction and dilation (8, 21). However, in the context of an immune-inflammatory response, a cascade of inflammatory mediators and reactive oxygen species is unleashed, leading to endothelial dysfunction (22, 23). This dysfunction manifests as reduced production of pulmonary vasodilators, increased production of pulmonary vasoconstrictors, and enhanced expression of proliferation-inducing factors, thereby elevating vascular tension and ultimately driving vascular remodeling (24). Chronic inflammatory aggregates and the formation of tertiary lymphoid organs (TLOs) (25, 26). TLOs, which structurally resemble lymph nodes, include specialized zones for T-cells with dendritic cells (DCs), organized B-cell clusters containing germinal centers, high endothelial venules, and lymphatic vessels (27). TLOs are thought to develop in response to sustained local immune activation and are considered a hallmark of chronic diseases (28). Within TLOs, tissue-migrated DCs present antigens to naïve T-cells, inducing their activation and differentiation (27). Immune cells such as T cells, B cells, and macrophages are activated, releasing inflammatory mediators that contribute to vascular remodeling and endothelial dysfunction.

In CTD-PAH, various pro-inflammatory molecules, such as interleukin (IL)-1, IL-6, tumor necrosis factor (TNF)-α and chemokines (such as chemokine ligand 2(CCL2)/monocyte chemoattractant protein-1 (MCP-1), RANTES chemokines or fracta) are synthesized by fibroblasts, ECs and vascular SMCs (9, 29, 30). In SSc-PAH, this pro-inflammatory signal involves oxidative stress and the production of a large number of pro-inflammatory molecules (31). For example, in SSc-PAH, autoreactive T cells infiltrate the pulmonary vasculature and secrete cytokines like interferon (IFN)-γ and IL-17, which promote smooth muscle cell proliferation and fibrosis (32). Furthermore, SLE patients with severe PAH exhibit enhanced expression of various growth factors and chemokines such as RANTES/CCL5 and fractalkine/fractalkine (CX3CL1) within the pulmonary artery, emphasizing the complex interplay of factors involved in this condition.

Notably, immunoglobulins and complement have been found to accumulate on arterial walls, triggering pulmonary vasculitis (33). The presence of these immune complexes within pulmonary vascular walls may contribute to the development of SLE-PAH (34). In SLE-PAH, immune complexes preferentially adhere to larger blood vessels, whereas in SLE-induced pneumonia, smaller vessels may be the primary sites of immune complex deposition (34). However, some researchers argue that inflammation appears to be less significant in the pathogenesis of SSc-PAH and MCTD-PAH, in contrast to SLE-PAH, where the features closely resemble the plexogenic lesions observed in IPAH (35). These variations in the inflammatory profile of SSc-PAH may account for the limited efficacy of immunosuppressive therapies in this condition. Additionally, genetic abnormalities are less common in CTD-PAH compared to IPAH, although they may still contribute in specific cases. An analysis of 79 CTD-PAH patients screened for a panel of 35 PAH-specific genes identified abnormalities in 9 individuals (11.4%) (36). Left ventricular dysfunction, prevalent in CTD, can result in pulmonary venous hypertension, particularly evident in SSc-PAH, where pulmonary veno-occlusive lesions are more pronounced (37).

## 3 DCs in CTD-PAH

DCs are effective and multifunctional antigen presenting cells, and their migration ability is the key to start protective pro-inflammatory and tolerant immune response (38). At the crossroads of innate immunity and adaptive immunity, dendritic cells do play a prominent role in the immune monitoring of self and non-self antigens and the initiation and coordination of specific adaptive immune responses of different types of antigens (39). Therefore, the first line of defense is very important at the barrier, especially in the lungs. However, they are also involved in the pathogenesis and progress of highly prevalent respiratory diseases (40).

Recent studies have demonstrated that DCs become activated and accumulate in the lungs of patients with CTD-PAH (27). These activated DCs enhance the production of inflammatory cytokines and chemokines, which in turn lead to pulmonary vascular remodeling and increased pulmonary vascular resistance. Additionally, the levels of inflammatory cytokines and chemokines produced by these DCs are elevated. In patients with SSc, circulating type 2 conventional DCs (cDCs) exhibit increased production of IL-6, IL-10, and tumor necrosis factor-α (TNF-α) following stimulation with TLR2 and TLR4 (41, 42). These cytokines are believed to play a crucial role in the immunopathology of PAH. Notably, IL-6 stands out as a critical cytokine in the pathogenesis of PAH, as evidenced by the development of pulmonary hypertension symptoms in mice overexpressing IL-6, whereas IL-6-deficient mice do not develop pulmonary hypertension under hypoxic conditions (43, 44). These findings indicate that DCs contribute to the development and progression of CTD-related PAH through their pro-inflammatory effects.

Plasmacytoid DCs (pDCs) are primarily found in lymphoid tissues and blood under normal conditions. The severity of lung diseases in SSc patients is related to the incidence of pDCs found in the lungs (45). Importantly, pDC plays a direct role in causing and maintaining fibrosis, because their consumption has been proved to improve pulmonary fibrosis. During inflammation, pDCs migrate to peripheral tissues, where they produce IFNs and facilitate the activation of immune cells. Several autoimmune diseases are associated with the interferon gene signature, to which different cells contribute. In patients with SLE and SSc, the number of circulating pDCs is reduced compared to healthy controls, likely due to their migration to affected tissues (46, 47). In SSc patients, elevated serum levels of C-X-C motif chemokine 10 (CXCL10) are linked with PAH, suggesting that pDCs may have a significant role in the immunopathology of the disease (48). Besides IFN, pDCs are also the primary producers of CXCL4 in SSc (49). The pDC of SSc patients abnormally expressed Toll-like receptor (TLR) 8, while TLR8 was not expressed in healthy conditions (47). This abnormal expression contributes to the disease progression, because the signal transduction through TLR8 will induce the production of CXCL4 (47). CXCL4 can attract CD45-positive cells into target tissues, potentially contributing to tissue remodeling and disease progression. In addition, the expression of TLR8 leads to the infiltration of pDCs into tissues, which aggravates the disease and leads to fibrosis (47). Activation of TLR9 under anoxic conditions has also been proved to induce the production of CXCL4 (50).

**Figure 1:**
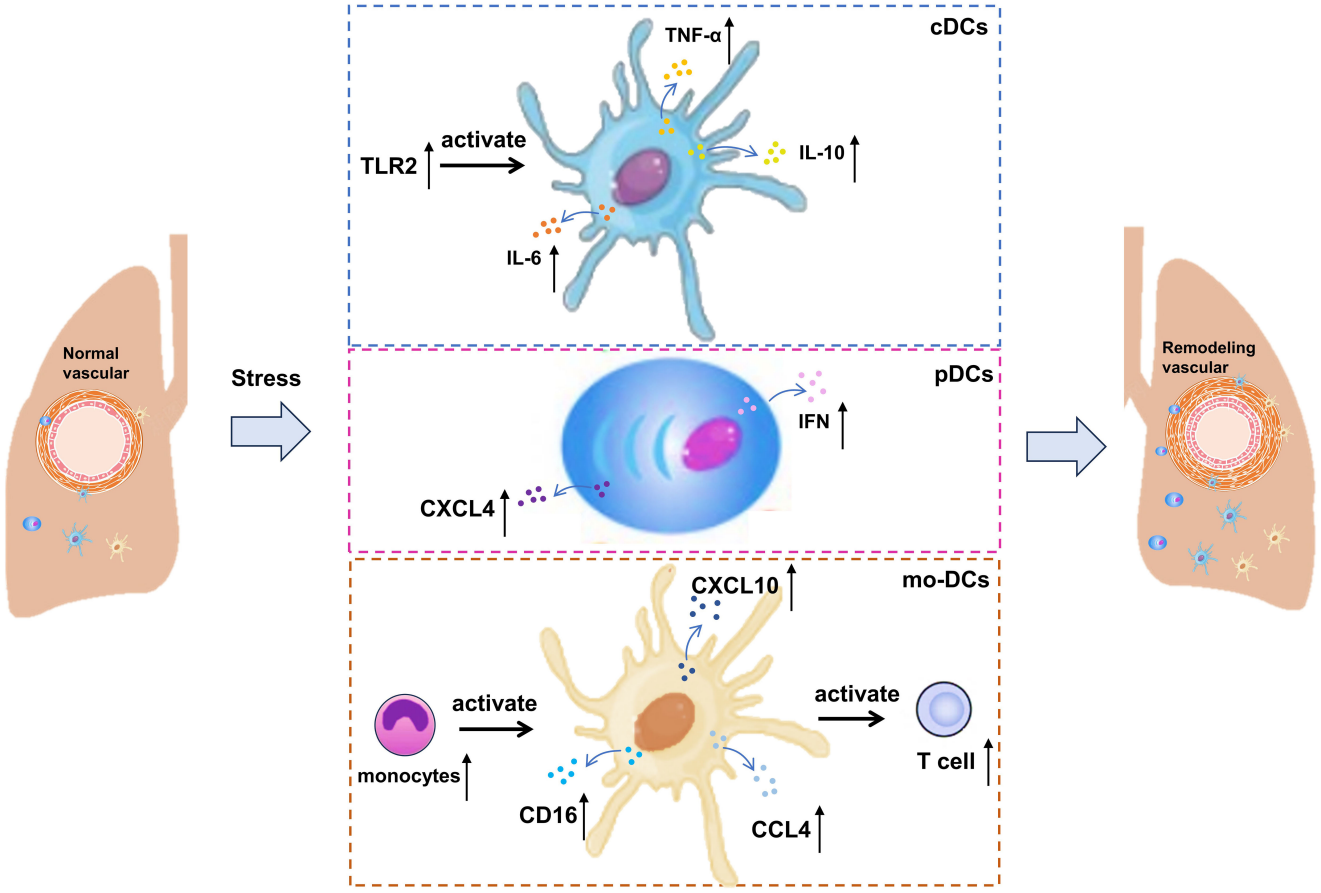
Involvement of cDCs, pDCs and mo-DCs in CTD-PAH. cDCs, circulating type 2 conventional; pDCs, plasmacytoid DCs; IL, interleukin; CCL, C-C motif chemokine ligand; TNF, tumor necrosis factor-alpha; CXCL, C-X-C motif chemokine ligand.

Additionally, monocytes serve as precursors for mo-DCs, which are produced under inflammatory conditions (51), and there is an observed increase in the number of non-classical monocytes in SSc-PAH (52). Non-classical monocytes, which express CD16, are known to monitor the endothelium for danger signals. They can differentiate into tissue-resident macrophages under steady-state conditions or into anti-inflammatory macrophages during inflammation to assist in tissue repair. At the same time, non-classical monocytes expressing CXCL10, CXCL8, and CCL4 are involved in SSc pathology, with higher numbers observed in SSc patients compared to controls (41). In summary, the increased pulmonary expression of chemokines may draw monocytes to the lungs of CTD-PAH patients, where they become activated and undergo gene expression changes due to the pro-inflammatory environment. These modified monocytes may then give rise to mo-DCs at the site of inflammation, capable of inducing T cell activation. The roles of cDCs, pDCs, mo-DCs and their inflammatory mediators in CTD-PAH are shown in **Figure 1**.

## 4 Lymphocytes in CTD-PAH

**Figure 2:**
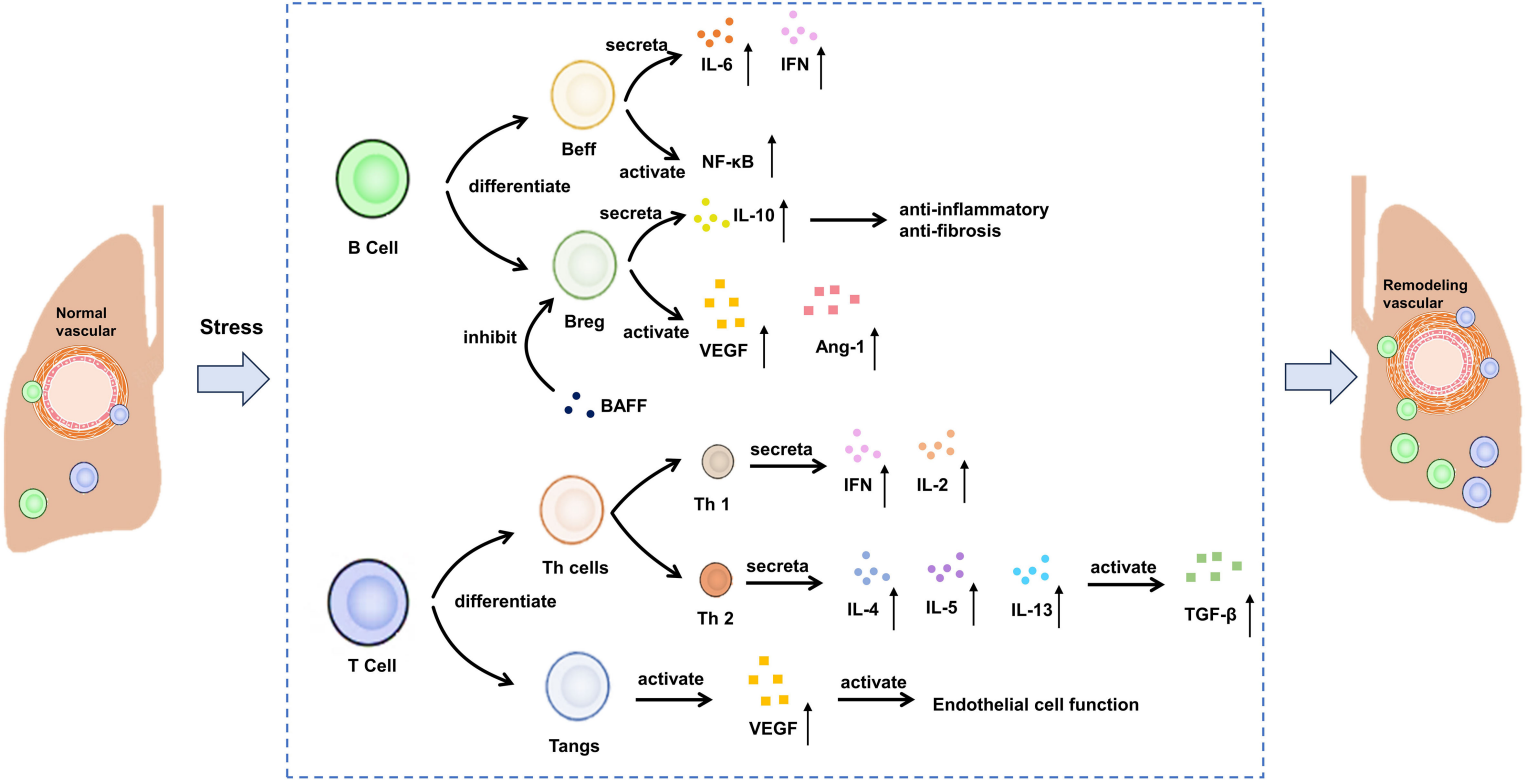
Potential mechanism of B cells and T cells on CTD-PAH. Beff: effector B cells, Breg, regulatory B cells; BAFF, B cell activating factor; VEGF, vascular endothelial growth factor; TGF-β, transforming growth factor-beta; IL, interleukin; IFN, interferon.

Lymphocytes are the main immune cells in the body, which are responsible for removing pathogens such as bacteria, viruses and parasites, thus protecting the human body from infection. They play a central role in the immune system by secreting cytokines, participating in cellular immunity and humoral immunity (53). The immune cells that mainly play a role include B cells and T cells, which participate in the immune activation in the process of vascular remodeling. The roles of c B cells, T cells and their inflammatory mediators in CTD-PAH are shown in **Figure 2**.

B cells have the ability to differentiate into plasma cells, which are responsible for producing autoantibodies. B cells achieve this goal by presenting antigen, producing various cytokines and promoting the differentiation of T effector cells (22, 54). B cells play a significant role in the formation of autoantibodies in SSc. In recent years, there has been growing recognition that B cells are a major source of pro-inflammatory cytokines, particularly IL-6 and IFN-γ, in autoimmune diseases (55). IL-6, a potent pro-inflammatory cytokine, also has strong fibrotic effects (56). For example, circulating B cells in SSc patients produce more IL-6 compared to healthy controls (57, 58). However, there is limited data on these pro-inflammatory characteristics in PAH. In a novel approach to studying SSc-PAH using female mice deficient in P-selectin glycoprotein ligand-1, IFN-γ-producing B cells showed greater lung infiltration compared to the control group (59). Additionally, in a rat model of PAH induced by the combination of anti-vascular endothelial growth factor (VEGF) Sugen-5416 injection and ovalbumin immunization, the depletion of B cells correlated with reduced IL-6 expression in the lung (60). Besides, for the peripheral blood mononuclear cells of SSc-PAH patients, the genes involved in B-cell receptor signaling and NF-κB pathway in the disease group were significantly up-regulated (61, 62). Unlike effector B cells (Beffs), regulatory B cell (Bregs) produce IL-10, an anti-inflammatory and anti-fibrotic cytokine (63). Furthermore, the role of serum B cell-activating factor (BAFF) has been well established in a murine model of SSc induced by bleomycin (64). BAFF inhibits Bregs and their ability to produce IL-10. In individuals with SSc-PAH, the levels of circulating CD24^hi^ CD27 Bregs are lower compared to SSc patients without PAH (65). While the existence and role of this subset in pulmonary arterial hypertension are yet to be thoroughly investigated, indirect evidence suggests a direct involvement of B cells in the vascular system of these patients (66). In SSc, B cells exhibit a higher tendency to produce vasculogenic mediators such as vascular endothelial growth factor and angiopoietin-1 compared to healthy controls, with no difference observed between patients with and without PAH (67).

T cells are an important part of adaptive immune response, including helper T cells (Th cells), regulatory T cells (Tregs) and angiogenic T cells (Tang), etc (68). Different types of T cells have specific functions and reactions in the inflammatory cascade reaction. Th cells produce a pro-inflammatory response, while Tregs exert a balanced response to achieve self-tolerance and prevent autoimmune (53). Similar to B cells, T cells can be categorized into two main opposing subpopulations: type 1 T cells, which primarily produce IFN-γ and IL-2, and type 2 T cells, which release IL-4, IL-5, and IL-13, thereby activating fibroblasts via the transforming growth factor (TGF)-β pathway (63, 69). An examination of T cell subpopulations in SSc has revealed a complex phenotype (70). Alongside Th2 cells, Th22, Th17, and CD4+ T cells reactive to topo-I play an active role in initiating pulmonary involvement in SSc (70, 71). Specifically, the topo-I-reactive CD4+ T cells demonstrate a Th17 phenotype and, along with Th22 cells, are elevated in patients, showing a negative correlation with pulmonary function parameters. Th17 cells produce IL-17, known for its fibrotic properties (72). At the same time, it was found that the expressions of IL-7R, LCK and HDAC1 were positively correlated with the number of T cell CD4 initiation and T cell CD4 memory. They reduce T cells in SSc-PAH PBMCs by regulating T cell activation (32). Although an increase in regulatory Tregs has been linked to decreased functional capacities in SSc, the precise role of these cells remains poorly understood (73). Studies have also delved into the involvement of T cells in angiogenesis. Hur et al. explored T cell subsets expressing CD31 and CXCR4, categorizing them as angiogenic T cells (Tang) due to their significant impact on vascular formation (74, 75). A recent study revealed their role in SSc pathogenesis. The presence of Tang cells is higher in SSc-PAH patients compared to those without PAH and healthy individuals (76). Moreover, there is a positive correlation between Tang cell numbers and VEGF levels in SSc-PAH, suggesting a connection between Tang cell activity and endothelial function.

## 5 Macrophages in CTD-PAH

Macrophages can remove pathogens and foreign bodies through their powerful phagocytosis, and serve as antigen presenting cells, presenting the treated antigens to T cells and B cells, thus initiating specific immune response (77). At the same time, macrophages can secrete a variety of inflammatory mediators, regulate immune response and promote inflammation regression (77). Macrophages play a crucial role in local innate immunity and provide comprehensive protection of the lungs against external substances (78). M1 macrophages are activated during the early inflammatory phase and induce tissue damage, with this differentiation pathway being regulated by damaged epithelial cells and IFN-γ (79). On the other hand, M2 macrophages, which exhibit fibrotic characteristics, are predominant during the proliferative phase (80–82). These activated macrophages can mitigate the differentiation of fibroblasts into myofibroblasts, a process particularly notable in SSc (82, 83). M2 macrophages, identifiable by their CD163+ and CD204+ markers, accumulate in the skin and serum of SSc patients (79). Additionally, M2 macrophages produce the chemokine CCL18, which can induce T cell migration and stimulate fibroblasts to produce collagen (84). Consequently, elevated levels of CCL18 in SSc patients are regarded as markers of lung fibrotic remodeling (85). While M2 cells are known for their fibrotic attributes, other cell populations also contribute to this complementary remodeling process. In fact, a mixed M1/M2 macrophage population is associated with SSc-PAH in both human and murine models (86, 87). Furthermore, alterations in macrophage-endothelial interactions can precipitate vascular pathologies and subsequent fibrosis. In models of bleomycin-induced injury, endothelial-derived cells exacerbate fibrosis and exhibit markers indicative of endothelial-mesenchymal transition (88). Notably, by knocking out RGC32, macrophage activation shifts from M2 to M1, which consequently reduces the skin and lung manifestations of bleomycin-induced pulmonary fibrosis (89). In addition, recent studies have shown that Regnase-1 is a multifunctional protein with RNAse activity, which can bind and degrade the mRNA of various inflammatory cytokines, thus inhibiting the inflammatory reaction. In patients with CTD-PAH, the expression level of Regnase-1 is decreased, which may lead to the over-expression of inflammatory cytokines, and then promote the development of pulmonary hypertension (90). The lack of Regnase-1 in macrophages will lead to the spontaneous development of severe CTD-PAH-like lesions in mice. This indicates that Regnase-1 in macrophages plays a key role in maintaining immune homeostasis and preventing the occurrence of CTD-PAH (90).

**Figure 3:**
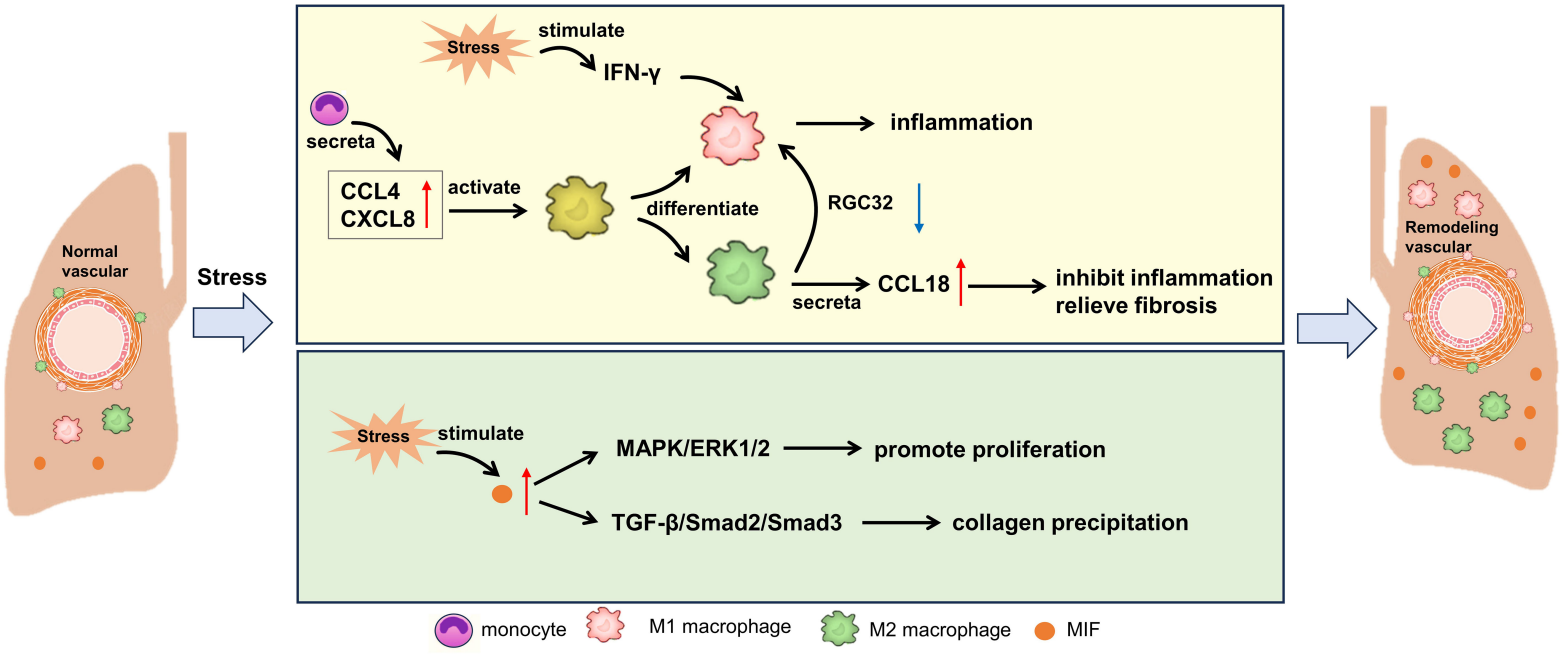
Potential mechanism of macrophages on CTD-PAH. MIF, macrophage migration inhibitory factor; CCL, C-C motif chemokine ligand; CXCL, C-X-C motif chemokine ligand; IFN, interferon; RGC32, response gene to complement 32; MAPK, mitogen-activated protein kinases; ERK, extracellular signal-regulated kinase; TGF, transforming growth factor.

Macrophage migration inhibitory factor (MIF) is a substance that can limit the activity of macrophages *in vivo*. Its main function is to limit the excessive movement of macrophages, promote the infiltration of macrophages in inflammatory sites, and participate in immune regulation (91). A study investigated the role of MIF in SLE-PAH (92). Circulating MIF levels were measured in SLE patients, SLE-PAH patients, and healthy donors. The results showed that circulating MIF was elevated in SLE-PAH patients compared to both SLE patients and healthy donors. In SLE mice, those with higher right ventricular systolic pressure (RVSP) produced more MIF protein in the pulmonary arteries than those with lower RVSP. Treatment with MIF098 reduced RVSP and inhibited excessive proliferation, muscularization, and collagen deposition in the distal pulmonary arteries of hypoxia-challenged mice. Additionally, MIF098 suppressed pulmonary arterial smooth muscle cell proliferation and migration by modulating the Mitogen-Activated Protein Kinase/Extracellular Signal-Regulated Kinase 1/2 signaling pathway and cell cycle-related proteins. In cell experiments, MIF098 also decreased collagen synthesis by inhibiting the TGF-β1/Smad2/Smad3 pathway. These findings suggest that MIF could serve as a biomarker and therapeutic target for SLE-PAH. MIF antagonists may be an effective means to improve SLE-PAH. The pathological mechanism involved above is shown in **Figure 3**.

## 6 Vascular cells in CTD-PAH

Immune cells can regulate angiogenesis by secreting cytokines such as VEGF and TNF-α. Immune cells can promote angiogenesis (such as tumor-associated macrophages) and inhibit angiogenesis (such as CD8+T cells) (93). Angiogenesis can affect the recruitment and infiltration of immune cells, and then affect the effect of immune response. Although vascular ECs are not professional antigen presenting cells, they can present antigens to T cells and express adhesion factors and cytokines to participate in immune response. ECs play a key role in maintaining vascular homeostasis under various stimuli, and regulate inflammation through mediators such as NO, ET, cell adhesion molecules, cytokines and chemokines (22). It is found that leptin derived from s plays a role in the immune pathogenesis of SSc-PAH by controlling regulatory T cells (94). At the same time, endothelial activation occurs in SSc, and Bosentan can block T cell/endothelial interaction in SSc-PAH and regulate the expression of vascular factors in serum (95). In addition, the researchers detected the response of human pulmonary artery ECs to BMPR2 signal and pyrophosphate factor stimulated by lipopolysaccharide. In PAECs interfered by autologous BMPR2+/R899X ECs and SIMPR 2, the expressions of IL-8 and E- selectin were up-regulated. The defect of BMPR2 signal transduction and proinflammatory factors promote vascular remodeling in SLE-PAH (96).

As an important vascular cell, fibroblasts also play an important role in CTD-PAH. Pulmonary fibrosis is a sign of patients with SSc-PAH, and fibroblasts are the main target cells in this process. Fibroblasts express TGF-β and platelet-derived growth factor receptor (97). Overregulation of Wnt/β-catenin signaling pathway (98) and increased expression of insulin-like growth factor binding protein regulate the induction of TGF-β in fibroblasts. All these overexpressed protein induce fibrosis by transforming fibroblasts into myofibroblasts (99). At the same time, the study showed that in TGF-β-dependent SSc-PAH mouse model, bone morphogenetic protein receptor (BMPR)2 decreased, signal transduction was damaged and receptor turnover activity changed. Similarly, the expression of BMPR2 was significantly decreased in SSc lung tissue and fibroblasts. The increase of proteasome degradation of BMPR2 seems to be the basis, which may be caused by the increase of TGF-β activity. This suggests that the damage of BMP signal transduction caused by the increase of TGF-β dependent receptor degradation may promote the susceptibility of PAH in SSc (100).

## 7 Immunosuppression therapy in CTD-PAH

At present, the clinical treatment of CTD-PAH includes specific treatment for PAH and treatment for primary CTD. The specific treatment for PAH is to use targeted drugs. For example, Bosentan can improve exercise tolerance, cardiac function classification, hemodynamic parameters and clinical deterioration time of patients with CTD-PAH (101). At the same time, the study confirmed that Bosentan can prevent endothelial activation in SSc by restoring T cell function (95). For the treatment of primary CTD, high-dose glucocorticoids (cyclophosphamide, mycophenolate mofetil, azathioprine, methotrexate or hydroxychloroquine, etc.) combined with immunosuppressants are usually used to alleviate the condition of CTD and effectively improve CTD-PAH (9). Different from all previous therapeutic drugs for PAH, Sotatercept is an activin signal inhibitor and a First-in-class activin receptor IIA-Fc (ActRIIA Fc) fusion protein, which can selectively bind TGF-β family ligands, restore the balance between pro-proliferation and anti-proliferation signal pathways related to pulmonary artery wall and right ventricular remodeling, and play the role of inhibiting cell proliferation, reversing vascular remodeling and smoothing blood vessels (102). It was found that the treatment with ActRIIA-Fc significantly reversed the expression of pro-inflammatory and proliferative genes and normalized macrophage infiltration in the lungs of diseased rodents (7). This shows that sotatercept may have anti-inflammatory activity besides its anti-proliferation effect on vascular cells.

Growing evidence confirms the significant involvement of the immune system in the occurrence and development of CTD-PAH. Some studies have explored the potential of immunosuppressive therapy as a treatment target for CTD-PH. Among these, rituximab (an anti-CD20 monoclonal antibody)-induced B cell depletion has been the most researched intervention. Patients treated with rituximab exhibited reductions in rheumatoid factor, IL-12, and IL-17 (103). Several reports indicated that CTD-PAH patients experienced improvements in conditions other than pulmonary vascular diseases following rituximab treatment (104–106). However, the role of immunosuppression in SSc-PAH remains unclear, as there has been no observed response to corticosteroid or cytotoxic therapies. The pathophysiological differences between SSc-PAH and other types of CTD-PAH may explain the varying responses to immunosuppressive treatments. A recent study by Zamanian et al. revealed that after 24 weeks of rituximab treatment, there was no significant change in the six-minute walk distance (6MWD) for SSc-PAH patients, although an improving trend was noted (103). The authors suggest that low levels of rheumatoid factor, IL-2, and IL-17 might predict a favorable response to rituximab. For non-SSc CTD-PAH patients, immunosuppressive therapy, such as glucocorticoids or macitentan, could be considered, especially if they present with non-cardiopulmonary manifestations, potentially benefiting from the treatment. Further research is essential to better understand the role of rituximab in specific SSc-PAH patients (11). Variability in study outcomes may be attributed to differences in sample sizes, leading to experimental errors. Moreover, during the research, the primary outcome measure was changed from hemodynamic improvement to 6MWD variability due to the unexpectedly low baseline pulmonary vascular resistance in SSc-PAH patients, which significantly reduced the utility of the original primary outcome measure (66). Interestingly, an independent reanalysis of trial data focused on identifying biomarker characteristics that could indicate rituximab efficacy within subgroups, uncovering noteworthy findings. It is also noteworthy that in studies using a pulmonary arterial hypertension animal model, anti-CD20 therapy began during disease induction, making it more of a preventive rather than a curative treatment (107, 108). Therefore, further research is required to determine the relevance and positioning of B-cell depletion in the PAH treatment arsenal.

The Bruton tyrosine kinase (BTK) inhibitor has shown promising results in improving hemodynamics, reducing right ventricular hypertrophy, and mitigating pulmonary arterial remodeling and fibrosis, as well as reversing endothelial-to-mesenchymal transition in PAH rats (109). BTK expression primarily co-localizes with macrophages, suggesting that the inhibitor’s effects are largely mediated through its action on macrophage BTK (109). Moreover, an increase in intracellular BTK in the B cells of CTD-PAH patients was associated with elevated serum autoantibodies (6). This indicates that BTK inhibition might alleviate PAH, at least in part, through its impact on B cells. In SSc patients, ibrutinib, a BTK inhibitor, has been found to reduce the production of pro-inflammatory cytokines and autoantibodies by peripheral B cells, while not affecting their IL-10 secretion (110). These findings suggest that BTK inhibitors could potentially serve as a therapeutic strategy for PAH by targeting both macrophages and B cells, thereby addressing multiple facets of the disease.

## 8 Conclusion and prospect

It is generally believed that autoimmune activation plays a key role in the pathophysiology of various subtypes of CTD-PAH. Their abnormal activation promotes the inflammatory environment and vascular remodeling characteristics of this devastating disease through various mechanisms, including autoantibody production, cytokine release and direct cell interaction. In the pathophysiology of CTD-PAH, the meaning of immune imbalance and immune cell activation is “enemy”, which leads to vascular cell damage and enlarges vascular inflammation. However, many molecular and cellular mechanisms behind this process remain unsolved. A better understanding of how immunity promotes the development of CTD-PAH is very important to promote the immunosuppressive treatment of this disease.
